# Online psychotherapy in times of COVID-19: professional’s experience

**DOI:** 10.1192/j.eurpsy.2021.921

**Published:** 2021-08-13

**Authors:** A. Jambrosic Sakoman, T. Jendricko, I. Zegura, M. Skelin, P. Brecic

**Affiliations:** 1 Department Of Psychotic Disorders, University Psychiatric Hospital Vrapče, Zagreb, Croatia; 2 Department Of Psychotherapy S, University Psychiatric Hospital Vrapče, Zagreb, Croatia; 3 Department For Affective Disorders, University Psychiatric Hospital Vrapče, Zagreb, Croatia

## Abstract

**Introduction:**

The global pandemic of corona virus has brought on the imperative of swift organisational changes within mental health care institutions. Outpatient psychiatric care system with all its complex features was organized online. Patient’s adjustment to different modality of treatment, and their benefit, was of our primary concern. Mental health professionals had many challenges in this process as well. Couple of months into providing this form of treatment, we were interested in therapist experience.

**Objectives:**

The aim of this study was to highlight challenges faced by the various mental health professionals that were a part of interdisciplinary team at University Psychiatric Hospital Vrapče.

**Methods:**

We used self-assessment scales to assess mental health professionals experience and to recognize some of the possible difficulties in this process. We have examined satisfaction with available technology, suitability of different psychotherapeutic background when providing online treatment, therapists competencies for this kind of treatment and levels of perceived stress. Mental health treatment comprised individual and group approach, and was provided by interdisciplinary teams of psychiatrists, clinical psychologists, social workers, social pedagogues, psychiatric nurses, and work therapists.

**Results:**

The results show differences between professional’s psychotherapy background, educational level and previous experience in perceived efficiency and experienced stress in the process of providing online treatment.

**Conclusions:**

The findings of this study have proven that professionals perceive technical, organisational standards, previous experience, and education to be important for their efficiency. This should be taken into account when developing standards in online treatment within mental health institutions.

**Conflict of interest:**

No significant relationships.

